# Sodium Butyrate Reduces *Salmonella* Enteritidis Infection of Chicken Enterocytes and Expression of Inflammatory Host Genes *in vitro*

**DOI:** 10.3389/fmicb.2020.553670

**Published:** 2020-09-16

**Authors:** Anamika Gupta, Mohit Bansal, Basanta Wagle, Xiaolun Sun, Narayan Rath, Annie Donoghue, Abhinav Upadhyay

**Affiliations:** ^1^Department of Poultry Science, University of Arkansas, Fayetteville, AR, United States; ^2^Poultry Production and Product Safety Research Unit, United States Department of Agriculture-Agriculture Research Station, Fayetteville, AR, United States; ^3^Department of Animal Science, University of Connecticut, Storrs, CT, United States

**Keywords:** primary chicken enterocytes, chicken macrophages, sodium butyrate, anti-inflammatory, *Salmonella*, gene expression

## Abstract

*Salmonella* Enteritidis (SE) is a facultative intracellular pathogen that colonizes the chicken gut leading to contamination of carcasses during processing. A reduction in intestinal colonization by SE could result in reduced carcass contamination thereby reducing the risk of illnesses in humans. Short chain fatty acids such as butyrate are microbial metabolites produced in the gut that exert various beneficial effects. However, its effect on SE colonization is not well known. The present study investigated the effect of sub-inhibitory concentrations (SICs) of sodium butyrate on the adhesion and invasion of SE in primary chicken enterocytes and chicken macrophages. In addition, the effect of sodium butyrate on the expression of SE virulence genes and selected inflammatory genes in chicken macrophages challenged with SE were investigated. Based on the growth curve analysis, the two SICs of sodium butyrate that did not reduce SE growth were 22 and 45 mM, respectively. The SICs of sodium butyrate did not affect the viability and proliferation of chicken enterocytes and macrophage cells. The SICs of sodium butyrate reduced SE adhesion by ∼1.7 and 1.8 Log CFU/mL, respectively. The SE invasion was reduced by ∼2 and 2.93 Log CFU/mL, respectively in chicken enterocytes (*P* < 0.05). Sodium butyrate did not significantly affect the adhesion of SE to chicken macrophages. However, 45 mM sodium butyrate reduced invasion by ∼1.7 Log CFU/mL as compared to control (*P* < 0.05). Exposure to sodium butyrate did not change the expression of SE genes associated with motility (*flgG, prot6E*), invasion (*invH*), type 3 secretion system (*sipB*, *pipB*), survival in macrophages (*spvB*, *mgtC*), cell wall and membrane integrity (*tatA*), efflux pump regulator (*mrr1*) and global virulence regulation (*lrp)* (*P* > 0.05). However, a few genes contributing to type-3 secretion system (*ssaV*, *sipA*), adherence (*sopB*), macrophage survival (*sodC*) and oxidative stress (*rpoS*) were upregulated by at least twofold. The expression of inflammatory genes (*Il1*β, *Il8*, and *Mmp9*) that are triggered by SE for host colonization was significantly downregulated (at least 25-fold) by sodium butyrate as compared to SE (*P* < 0.05). The results suggest that sodium butyrate has an anti-inflammatory potential to reduce SE colonization in chickens.

## Introduction

*Salmonella* Enteritidis (SE) is one of the major bacterial pathogens responsible for causing food borne illnesses in humans (Kohli et al., 2018). Contaminated poultry meat and eggs are the major sources of SE infection in humans (Kollanoor-Johny et al., 2012). Hoffmann and Anekwe (2015) reported that non-typhoidal *Salmonella* ranked first in annual cost of illness and cause an annual economic burden of $4.43 billion per year impacting government and food industry. The incidence of *Salmonella* infection has been reported to have increased by ∼9% in 2018 as compared to 2015–2017 data (Marder et al., 2018). Despite the implementation of various interventions, SE remains a major cause of outbreaks related to consumption of contaminated poultry products (Antunes et al., 2016). In addition to conventional poultry systems, SE has also been isolated from backyard flocks (Kauber et al., 2017; Pal et al., 2017). The U.S. Centers for Disease Control and Prevention (CDC) recently reported multistate *Salmonella* outbreaks linked with backyard poultry in which 1003 people were infected from 49 states resulting in 175 hospitalizations and 2 deaths (CDC, 2018). Moreover, among 1.2 million illnesses annually, approximately 100,000 infections are due to antibiotic resistance of *Salmonella* against potential drugs such as ceftriaxone and ciprofloxacin which causes an annual illness of 33,000 and 36,000 respectively in the United States (Nair et al., 2018). *Salmonella* isolates resistant to five or more classes of antibiotics have been previously reported (CDC, 2013) further raising public health concerns.

Chickens disseminate SE in the environment by acting as asymptomatic carrier for the pathogen (Upadhyaya, 2015). The SE predominantly colonizes in the cecum of chickens thereby leading to contamination of carcasses during slaughter. Despite the presence of significant defensive barriers in the intestine, *Salmonella* has developed several strategies to colonize the gastrointestinal tract and penetrate the intestinal epithelium of humans and chickens. *Salmonella* interacts with the host (both humans and chickens) through an array of different bacterial proteins which contribute to invasion of intestinal epithelium (Lhocine et al., 2015). In the intestinal lumen, *Salmonella* uses flagella and fimbriae for cell attachment (Gart et al., 2016) and adhesion proteins such as SiiE and BapA to attach to the intestinal epithelium (Fàbrega and Vila, 2013). *Salmonella* injects bacterial effector proteins such as SipA, SopA, SopB, SopD, and SopE2 into host epithelial cells by utilizing Type III secretion systems (T3SS) encoded on *Salmonella* Pathogenicity Island-1 (SPI-1) for cytoskeletal rearrangement and bacterial engulfment (Wemyss and Pearson, 2019). SPI-1 plays a crucial role in colonization and invasion of *Salmonella* into host intestinal epithelial cells which further induces secretion of inflammatory cytokines and antimicrobial peptides such as IL-1β, IL-12, IL-18, α-defensins, and cathelicidins along with activation of macrophages and recruitment of neutrophils (Gart et al., 2016). Therefore, *Salmonella* induces an inflammatory immune response in host intestine which allows it to compete with commensal microbiota and to effectively colonize in the gut (Hallstrom and McCormick, 2011; Fàbrega and Vila, 2013; Gart et al., 2016).

In addition, evidence exists that SE survives in chicken macrophages and disseminates systemically thereby contaminating meat and eggs (Foley et al., 2013). Internalization of *Salmonella* causes formation of *Salmonella* containing vacuoles (SCVs) which induces expression of Type III secretion systems (T3SS) encoded on *Salmonella* Pathogenicity Island-2 (SPI-2) through various effector proteins for bacterial replication and intracellular survival such as SpiC, PipB, SseJ, SifA, SspH, and SopD2 (Ly and Casanova, 2007; Foley et al., 2013; Wemyss and Pearson, 2019). *Salmonella* interferes with NADPH oxidase complex inside the phagocytic macrophages, which prevents superoxide production and allows the bacteria to survive inside macrophage cells.

Different approaches have been investigated to reduce SE colonization in chickens which include feeding competitive exclusion bacteria (Stern et al., 2001; Revolledo et al., 2006; Kim et al., 2020) antibiotics (Chadfield and Hinton, 2004), bacteriophages (Fiorentin et al., 2005; Atterbury et al., 2007; Nabil et al., 2018), vaccines (Inoue et al., 2008; Bearson et al., 2019; Wilde et al., 2019), plant derived compounds (Kollanoor-Johny et al., 2012; Upadhyaya et al., 2013, 2015; Darre et al., 2014; Johny et al., 2017), and organic acids (Xiong et al., 2016; Wu et al., 2018). However, limited antimicrobial efficacy, toxicity, palatability concerns, or adverse effect on production parameters necessitates the exploration of new antimicrobial compound for controlling *Salmonella* colonization in chickens.

Short chain fatty acids such as butyrate are microbial metabolites synthesized from fermentation of dietary fibers in the colonic lumen. Previous studies have shown that invasion of SE is influenced by short chain fatty acids in avian intestinal epithelial cells (Van Immerseel et al., 2003) and partially protected sodium butyrate based feed additives and coated forms of butyric acid reduced SE colonization in broiler chickens (Van Immerseel et al., 2005; Fernández-Rubio et al., 2009). Butyrate treatment also induces antimicrobial host defense peptides gene expression in the intestinal tract of chickens (Sunkara et al., 2011). Zhou et al. (2014) showed that butyrate treatment reduced the expression of inflammatory cytokines in chicken macrophages stimulated with LPS. In a recent study, the supplementation of dietary sodium butyrate in feed promoted growth, intestinal development by increasing length of villi in ileum with mucus secretion and improved morphological structure and biological function in broiler chickens (Wu et al., 2018). In addition, sodium butyrate at higher doses (800 mg/kg) modulates antioxidant capacity, decreased malondialdehyde concentration in the jejunal mucosa by regulation of intestinal microbial community in broilers chickens (Wu et al., 2018). Despite the multiple beneficial effects of butyrate, there is limited understanding on the efficacy of butyrate in reducing SE infection in chickens and its potential mechanism(s) of action.

Therefore, the objective of this study was to investigate the effect of sodium butyrate on SE adhesion and invasion of primary chicken enterocytes and macrophages. In addition, the effect of sodium butyrate on the expression of virulence genes of SE and inflammatory genes in chicken macrophages challenged with SE infection was investigated.

## Materials and Methods

Our overall aim of this project was to investigate the effect of sodium butyrate on the virulence of SE and the response of the host *in vitro*. We hypothesized that sodium butyrate will reduce the virulence of SE and will modulate the host response to provide protection to the host against SE. The effect of sodium butyrate on SE was studied in two steps. First, the effect of sodium butyrate on the capacity of SE to attach and invade chicken primary enterocytes and macrophages was investigated using standard cell culture assay. Second, the effect of sodium butyrate on the expression of virulence genes of SE was studied using real-time qPCR. Similarly, the effect of sodium butyrate on the expression of inflammatory genes of chicken macrophages was studied using real-time qPCR. Details are provided below.

### Primary Chicken Enterocytes Cell Culture

The primary chicken enterocytes were cultured as described previously (Rath et al., 2018). Briefly, six, day-old male broiler chicks (Cobb 500) were obtained from Cobb-Vantress, Fayetteville, AR and were housed overnight (brooding temperature of ∼90°F with *ad libitum* water) as approved by Institutional Animal Care and Use Committee, University of Arkansas. Chicks were euthanized by cervical dislocation and small intestines were collected in a petri-dish containing Dulbecco’s modified minimum essential medium (DMEM F-12; HiMedia Laboratories Pvt., Ltd., Mumbai, India) enriched with 1X antibiotic antimycotic solution (Sigma-Aldrich, St Louis, MO, United States), 1X sodium pyruvate solution (Sigma-Aldrich), gentamicin solution (Sigma-Aldrich), 10 mM glutamine solution (Thermo Fisher Scientific, Carlsbad, CA, United States). Intestinal segments from the six chicks were pooled and rinsed three times with DMEM F-12 and squeezed to harvest villi from intestinal segments in petri plate containing DMEM F-12 medium. Harvested intestinal villi were centrifuged at 300 g for 10 min to form a pellet. The pellet was resuspended in 0.1% Streptomyces hyaluronidase (Sigma-Aldrich) and incubated for 60 min at 37°C in a humidified 5% CO_2_ incubator. The intestinal villi were centrifuged at 300 g for 10 min and further digested with 0.025% Trypsin: cell dissociation solution (Sigma-Aldrich) in the ratio of 1:9 for 15 min at 37°C in a humidified 5% CO_2_ incubator. Dissociated cells were layered over Histopaque-1119 (Sigma-Aldrich) for 30 min at 400 g for density gradient centrifugation. Cell layer at the interface of gradient medium was collected, suspended in DMEM F-12 and centrifuged at 300 g for 10 min. Cell clusters were resuspended in DMEM F-12 culture medium containing growth factors such as 10% heat inactivated fetal bovine serum (Thermo Fisher Scientific), 1X Insulin Transferrin Selenium (Sigma-Aldrich), 1X Epithelial cell growth supplement (EpiCGS, Sigma-Aldrich), 20 ng/mL epidermal growth factor (EGF, Thermo Fisher Scientific) for 24–48 h in a humidified 5% CO_2_ incubator at 37°C till it reached semi-confluency. Enterocytes were dissociated with Accutase (Sigma-Aldrich) to perform cell culture assays.

Chicken macrophages (HTC cells; a naturally transformed cell line) were cultured in Roswell Park Memorial Institute (RPMI) 1640 media (Thermo Fisher Scientific) (Rath et al., 2003) containing 10% fetal bovine serum, 1X antibiotic antimycotic solution, 1X sodium pyruvate solution, gentamicin solution, 10 mM glutamine solution at 37°C for 24–48 h in a humidified 5% CO_2_ incubator. The cells were cultured to semi-confluency of 50% followed by dissociation with Accutase to perform appropriate cell culture assays.

### Bacterial Strains and Culture Conditions

The SE strain GFP 338 has been previously used to study the differential response of macrophages (Sheela et al., 2003; Okamura et al., 2005) where it displayed significant intracellular viability. SE GFP 338 strain was isolated from egg associated food outbreaks by the Food and Drug Administration (Laurel, Md.) This strain was transformed with GFP containing plasmid that allows constitutive production of GFP under the control of a lac promoter. Therefore, we selected this strain for our study. SE was cultured in 10 mL of tryptic soy broth (TSB; Hardy Diagnostics CRITERION^TM^, Santa Maria, CA, United States) at 37°C for 18 h. Following subculture in 10 mL TSB for another 10 h, the culture was centrifugated at 2500 g for 10 min. The pellet was suspended in sterilized phosphate buffer saline (PBS, pH 7) and used as the inoculum. The enumeration of SE counts in inoculum was made by plating serial fivefold dilutions on brilliant green agar (BGA; Difco Laboratories, Detroit, MI, United States) and the plates were incubated at 37°C for 24 h for bacterial enumeration.

### Determination of Sub-Inhibitory Concentrations of Sodium Butyrate

The sub-inhibitory concentrations (SICs) of sodium butyrate against SE was determined as described previously (Upadhyaya et al., 2015). Briefly, twofold dilutions of sodium butyrate (363, 181.5, 90.75, 45, 22, and 11 mM) in TSB were prepared in sterile 96-well polystyrene tissue culture plate. The SE (∼6.0 Log CFU) was added to each well except negative controls and the plate was incubated at 37°C for 24 h under aerobic condition. The growth of SE was determined by measuring absorbance using spectrophotometric microplate reader (Benchmark; Bio-Rad Laboratories, Hercules, CA, United States) at 570 nm. The two highest concentrations of sodium butyrate that did not inhibit SE growth after 24 h of incubation were determined as the *SIC* for the present study.

### The Effect of SICs of Sodium Butyrate on Cell Viability of Primary Chicken Enterocytes and Chicken Macrophages

The effect of SICs of sodium butyrate on cell viability was performed as per standard protocol using MTT assay (Jung et al., 2005; Sakurazawa and Ohkusa, 2005). Primary chicken enterocytes and chicken macrophages were grown (10^4^ cells per well) using 96 well plate for 24 h at 37°C in a humidified, 5% CO_2_ incubator. Monolayers of the chicken enterocytes or chicken macrophages were incubated with or without (control) SICs of sodium butyrate for 2 h at 37°C in a humidified, 5% CO_2_ incubator and the MTT assay was performed as described above.

### The Effect of SICs of Sodium Butyrate on SE Adhesion to and Invasion of Chicken Enterocytes and Chicken Macrophages

The effect of SICs of sodium butyrate on SE adhesion to and invasion of primary chicken enterocytes cell culture and chicken macrophages was performed using attachment and invasion assays as described earlier (Wagle et al., 2017) with minor modifications. Primary chicken enterocytes or chicken macrophages (10^5^ cells per well) were seeded into 6-well plates (Costar) containing DMEM F-12 with 10% FBS and incubated for 48 h at 37°C in a humidified, 5% CO_2_ incubator to form a monolayer. A mid-log phase (10 h) culture of SE was inoculated on the primary chicken enterocytes and chicken macrophages (∼6 Log CFU/mL; multiplicity of infection 10:1) in the presence or absence of SICs of sodium butyrate. For the adhesion assay, an infected monolayer was incubated for 2 h followed by rinsing with PBS three times. The cells were lysed by treating with 0.1% Triton-X 100 for 20 min. The number of adhered SE was determined by dilution and plating of cell lysate on BGA plates followed by incubation at 37°C for 24 h under aerobic condition.

For the invasion assay, infected monolayers after an incubation of 2 h with SE were rinsed with PBS three times, followed by incubation with gentamicin (100 μg/mL, Sigma-Aldrich) at 37°C in a humidified, 5% CO_2_ incubator for additional 2 h to kill the extracellular bacteria. The cells were washed with PBS three times and lysed by 0.1% Triton-X 100. The cell lysate was diluted and plated on BGA plates for enumeration of invaded SE.

### The Effect of *SIC* of Sodium Butyrate on the Expression of Virulence Genes of SE

The effect of *SIC* of sodium butyrate on the expression of SE virulence genes was determined using real-time quantitative PCR (RT-qPCR) as described previously (Upadhyaya et al., 2015; Sun and Jia, 2018; Bansal et al., 2019). SE was cultured to mid-log phase with or without *SIC* of sodium butyrate in TSB at 37°C for 10 h. The total RNA was extracted using TRIzol reagents (Thermo Fisher Scientific) as per manufacturer’s protocol. Complementary DNA (cDNA) was synthesized using M-MLV kit (Thermo Fisher Scientific). Messenger RNA (mRNA) expression of SE genes was determined using SYBR Green PCR Master mix (Bio-Rad Laboratories, Inc., CA, United States) in a 384-well real-time PCR System (Model 7500 Fast Step One Plus system-Applied Biosystems, Thermo Fisher Scientific) and normalized to endogenous control, *16S* rRNA. The primers used in this study (Upadhyaya et al., 2015) were obtained from Integrated DNA Technologies, Inc. (Coralville, IA) (Table 1). After the thresholds (Ct) were obtained, the relative gene expression was calculated using 2^–Δ^ ^ΔCt^ method according to these reports.

**TABLE 1:** List of primers used for RT-qPCR analysis of SE genes.

Genes	Function	Primer	Sequence (5′–3′)
*flgG*	Motility	Forward	5′-GCGCCGGACGATTGC-3′
		Reverse	5′-CCGGGCTGGAAAGCATT-3′
*prot6E*	Motility	Forward	5′-GAACGTTTGGCTGCCTATGG-3′
		Reverse	5′-CGCAGTGACTGGCATCAAGA-3′
*fimD*	Motility	Forward	5′-CGCGGCGAAAGTTATTTCAA-3′
		Reverse	5′-CCACGGACGCGGTATCC-3′
*invH*	Invasion	Forward	5′-CCCTTCCTCCGTGAGCAAA-3′
		Reverse	5′-TGGCCAGTTGCTCTTTCTGA-3′
*sipB*	Type 3 secretion system	Forward	5′-GCCACTGCTGAATCTGATCCA-3′
		Reverse	5′-CGAGGCGCTTGCTGATTT-3′
*pipB*	Type 3 secretion system	Forward	5′-GCTCCTGTTAATGATTTCGCTAAAG-3′
		Reverse	5′-GCTCAGACTTAACTGACACCAAACTAA-3′
*orf245*	Type 3 secretion system	Forward	5′-CAGGGTAATATCGATGTGGACTACA-3′
		Reverse	5′-GCGGTATGTGGAAAACGAGTTT-3′
*sipA*	Type 3 secretion system	Forward	5′-CAGGGAACGGTGTGGAGGTA-3′
		Reverse	5′-AGACGTTTTTGGGTGTGATACGT-3′
*ssaV*	Type 3 secretion system	Forward	5′-GCGCGATACGGACATATTCTG-3′
		Reverse	5′-TGGGCGCCACGTGAA-3′
*spvB*	Survival in macrophages	Forward	5′-TGGGTGGGCAACAGCAA-3′
		Reverse	5′-GCAGGATGCCGTTACTGTCA-3′
*mgtC*	Survival in macrophages	Forward	5′-CGAACCTCGCTTTCATCTTCTT-3′
		Reverse	5′-CCGCCGAGGGAGAAAAAC-3′
*sodC*	Survival in macrophages	Forward	5′-CACATGGATCATGAGCGCTTT-3′
		Reverse	5′-CTGCGCCGCGTCTGA-3′
*tatA*	Cell wall and cell membrane integrity	Forward	5′-AGTATTTGGCAGTTGTTGATTGTTG-3′
		Reverse	5′-ACCGATGGAACCGAGTTTTTT-3′
*hflK*	Cell wall and cell membrane integrity	Forward	5′-AGCGCGGCGTTGTGA-3′
		Reverse	5′-TCAGACCTGGCTCTACCAGATG-3′
*ompR*	Cell wall and cell membrane integrity	Forward	5′-TGTGCCGGATCTTCTTCCA-3′
		Reverse	5′-CTCCATCGACGTCCAGATCTC-3′
*mrr1*	Efflux pump regulator	Forward	5′-CCATCGCTTCCAGCAACTG-3′
		Reverse	5′-TCTCTACCATGAACCCGTACAAATT-3′
*lrp*	Virulence regulation	Forward	5′-TTAATGCCGCCGTGCAA-3′
		Reverse	5′-GCCGGAAACCAAATGACACT-3′
*sopB*	Adherence	Forward	5′-GCGTCAATTTCATGGGCTAAC-3′
		Reverse	5′-GGCGGCGAACCCTATAAACT-3′
*xthA*	Exo/endonuclease activity	Forward	5′-CGCCCGTCCCCATCA-3′
		Reverse	5′-CACATCGGGCTGGTGTTTT-3′
*rpoS*	Oxidative stress	Forward	5′-TTTTTCATCGGCCAGGATGT-3′
		Reverse	5′-CGCTGGGCGGTGATTC-3′
*ssrA*	Metabolism	Forward	5′-CGAGTATGGCTGGATCAAAACA-3′
		Reverse	5′-TGTACGTATTTTTTGCGGGATGT-3′
*rfbH*	Lipopolysaccharide biosynthesis	Forward	5′-ACGGTCGGTATTTGTCAACTCA-3′
		Reverse	5′-TCGCCAACCGTATTTTGCTAA-3′
*16S*	16S rRNA	Forward	5′-CCAGGGCTACACACGTGCTA-3′
		Reverse	5′-TCTCGCGAGGTCGCTTCT-3′

### The Effect of SICs of Sodium Butyrate on the Expression of Inflammatory Genes of Chicken Macrophages Challenged With SE

The effect of sodium butyrate on the expression of inflammatory cytokine genes in chicken macrophages was performed using RT-qPCR, as described earlier (Sun and Jobin, 2014). Chicken macrophages (5 × 10^5^ cells per well) were seeded in 6-well plate and incubated at 37°C in a humidified, 5% CO_2_ incubator for 48–72 h. A mid-log SE culture was inoculated on HTC cells (∼6 Log CFU/mL; multiplicity of infection 10:1) in presence or absence of SICs of sodium butyrate followed by incubation for 4 h at 37°C in a humidified, 5% CO_2_ incubator. Following incubation, total RNA was isolated from chicken macrophages using TRIzol reagents (Thermo Fisher Scientific) as per manufacturer’s protocol. Complementary DNA (cDNA) was synthesized using M-MLV kit (Thermo Fisher Scientific). Messenger RNA (mRNA) expression of inflammatory mediators was determined using SYBR Green PCR Master Mix (Bio-Rad Laboratories, Inc., CA, United States) in a 384-well RT-qPCR System and normalized to endogenous control, *Gapdh*. The primers of each gene were designed from Primer 3 software (National Center for Biotechnology Information, Bethesda, MD) and obtained from Integrated DNA Technologies, Inc. (Coralville, IA) (Table 2).

**TABLE 2:** List of primers used for RT-qPCR analysis of host immune response genes.

Genes	Primer	Sequence (5′–3′)
*Il1*β	Forward	5′-GCATCAAGGGCTACAAGCTC-3′
	Reverse	5′-CAGGCGGTAGAAGATGAAGC-3′
*Il8*	Forward	5′-CCTCCTGCCTCCTACATTCA-3′
	Reverse	5′- ATCTCCAGCTCCTTTCACGA-3′
*Mmp9*	Forward	5′-CCAAGATGTGCTCACCAAGA-3′
	Reverse	5′-CCAATGCCCAACTTCTCAAT-3′
*Il12*α	Forward	5′-CAAACGAGGCACTCCTGAAG-3′
	Reverse	5′-GGTCTTCGTAGATCCCCTGC-3′
*Il12*β	Forward	5′-CTGATGAAGCACTGCCAGTTTAC-3′
	Reverse	5′-AAAGCGTGGACCACTCACTC-3′
*Il18*	Forward	5′-TTGCTTGTGGTTCGTCCAGA-3′
	Reverse	5′-GCTGAATGCAACAGGCATCC-3′
*Nos2*	Forward	5′-AAACTTCATCCCCCAACCAGC-3′
	Reverse	5′-GTTTCTAGTCGGGCCAGGTG-3′
*Il6*	Forward	5′-TTCCCCAGGTGGGAGGAATTG-3′
	Reverse	5′-ACAGCCACATCAAAATAGGCGA-3′
*Il10*	Forward	5′-AGCCTTCACCTTGATGGAGC-3′
	Reverse	5′-TGATGGGTAGTGAGGAGGGG-3′
*Gapdh*	Forward	5′-GACGTGCAGCAGGAACACTA-3′
	Reverse	5′-CTTGGACTTTGCCAGAGAGG-3′

*Il, Interleukin; Mmp9, Matrix metalloproteinase 9; Nos2, Nitric oxide synthase 2; Gapdh, Glyceraldehyde 3-phosphate dehydrogenase.*

### Statistical Analyses

The CFU counts of SE were logarithmically transformed (Log CFU) to maintain homogeneity of variance (Byrd et al., 2001). For all assays, triplicate samples were used, and the experiment was repeated two times. For cell culture assays and RT-qPCR gene expression for host immune response data were analyzed using One-way ANOVA in Graph-pad 7 Software. Treatment means were separated by Tukey’s multiple comparisons test. The changes in expression of SE genes in response to sodium butyrate were analyzed by using Student’s *t*-test for comparisons between treatment and controls. Probability of *P* < 0.05 was set for statistical significance.

## Results

### Sub-Inhibitory Concentrations of Sodium Butyrate Against SE

Based on growth curve analysis (12 h of SE incubation with sodium butyrate at 37°C), the three concentrations of sodium butyrate that did not inhibit growth of SE as compared to control were 11, 22 and 45 mM (*P* > 0.05; Data not shown). We selected the two highest SICs (22 and 45 mM) of sodium butyrate for further studies.

### The Effect of SICs of Sodium Butyrate on Cell Viability of Primary Chicken Enterocytes and Chicken Macrophages

**FIGURE 1:**
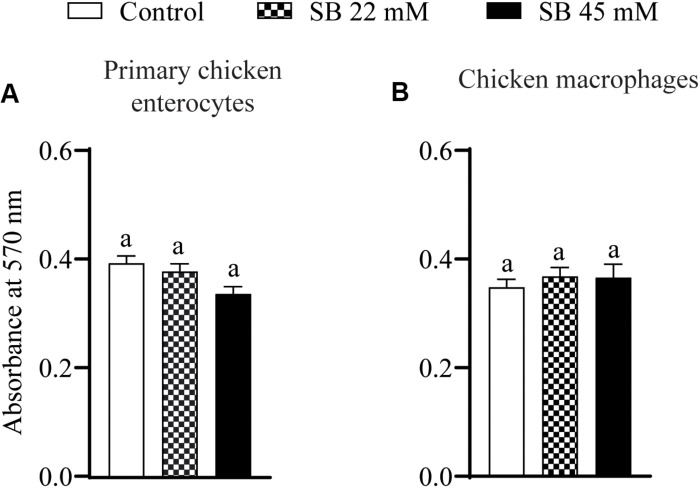
The effect of SICs of SB on cell viability of primary chicken enterocytes **(A)** and chicken macrophages **(B)** using MTT assay. Control represents enterocyte and macrophage cells not exposed to SICs of SB. Data presented as mean absorbance and error bar represents SEM (*n* = 6). Bar with different letters represents a statistical difference at *P* < 0.05.

The effect of SIC’s of sodium butyrate on cell viability of primary chicken enterocytes and chicken macrophages is shown in Figure 1. The control had an absorbance of ∼0.5 in chicken enterocytes and presence of two SICs of sodium butyrate does not affect cell viability (Figure 1A; *P* > 0.05). Similar results were observed with chicken macrophages wherein the presence of SICs of sodium butyrate did not significantly affect the viability of chicken macrophages (Figure 1B).

### The Effect of SICs of Sodium Butyrate on SE Adhesion to and Invasion of Primary Chicken Enterocytes and Chicken Macrophages

**FIGURE 2:**
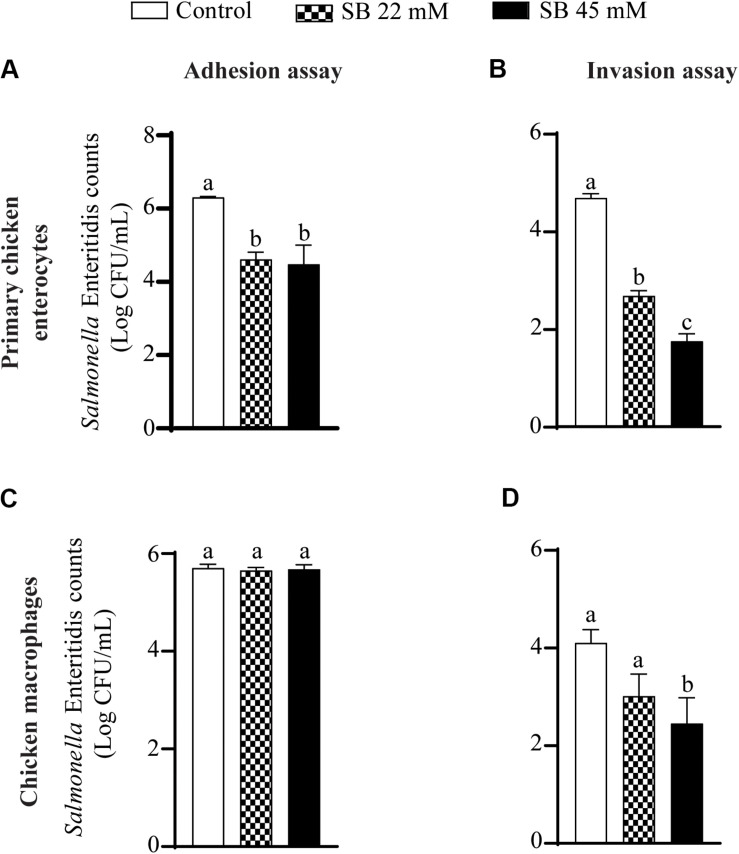
The effect of SICs of SB on SE adhesion **(A,C)** to and invasion **(B,D)** of primary chicken enterocytes **(A,B)** and chicken macrophages **(C,D)**. Data presented as mean Log CFU/mL and error bars represents SEM (*n* = 6). Bars with different letters represents a significant difference at *P* < 0.05.

Figure 2 shows the effect of SICs of sodium butyrate on SE adhesion to and invasion of primary chicken enterocytes and chicken macrophages. Approximately 6.3 Log CFU/mL SE adhered on primary chicken enterocytes (Figure 2A). The SICs (22 and 45 mM) of sodium butyrate significantly reduced adhesion of SE to primary chicken enterocytes by ∼1.7 and ∼1.8 Log CFU/mL, respectively, as compared to control. Similarly, the invaded SE counts in controls were ∼5 Log CFU/mL and the two SICs (22 and 45 mM) of sodium butyrate reduced invasion of SE by ∼2 and 2.93 Log CFU/mL, respectively (*P* < 0.05) (Figure 2B). In the chicken macrophages, ∼6 Log CFU/mL SE adhered (Figure 2C) and ∼4 Log CFU/mL invaded the cells (Figure 2D). The presence of 22 mM sodium butyrate did not reduce SE adhesion to and invasion of chicken macrophages (*P* > 0.05). In contrast to 22, 45 mM sodium butyrate significantly reduced invasion of SE by ∼1.7 Log CFU/mL as compared to controls (*P* < 0.05).

### The Effect of Sodium Butyrate on the Expression of SE Virulence Genes

**FIGURE 3:**
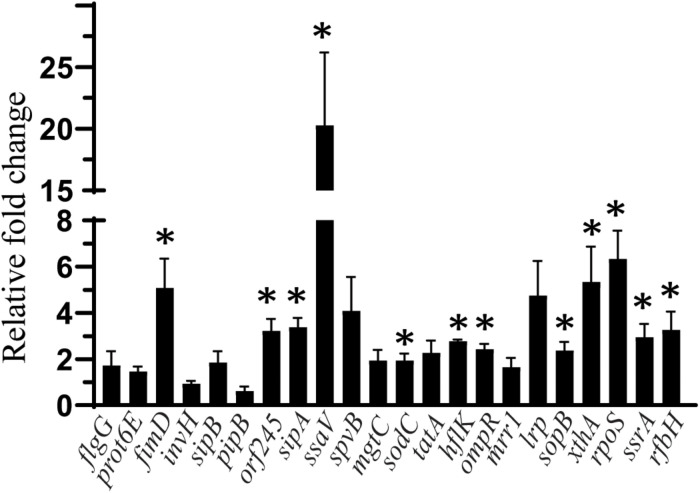
The effect of *SIC* 45 mM of SB on the expression of SE genes crucial for virulence and intestinal colonization. Data presented as relative fold change normalized to endogenous control, *16S* rRNA. * indicates significant change in the expression of genes at *P* < 0.05.

The effect of *SIC* (45 mM) of sodium butyrate on the expression of SE genes essential for virulence and intestinal colonization is shown in Figure 3. The expression of SE genes crucial for motility (*flgG, prot6E*), invasion (*invH*), type 3 secretion system (*sipB*, *pipB*), survival in macrophages (*spvB*, *mgtC*), cell wall and membrane integrity (*tatA*), efflux pump regulator (*mrr1*), and global virulence regulation (*lrp*) was not affected by sodium butyrate (*P* > 0.05). However, few genes contributing to type 3 secretion system components (*ssaV, orf245, sipA*), adherence (*sopB*), motility (*fimD*), exo/endonuclease activity (*xthA*), and oxidative stress (*rpoS*) were upregulated (*P* < 0.05). Similarly, genes such as *hflK* and *ompR* important for integrity of cell wall and cell membrane, metabolism (*ssrA*), macrophage survival (*sodC*), and lipopolysaccharide biosynthesis (*rfbH*) were slightly upregulated by sodium butyrate treatment.

### The Effect of Sodium Butyrate on the Expression of Inflammatory Genes in Chicken Macrophages Challenged With SE

**FIGURE 4:**
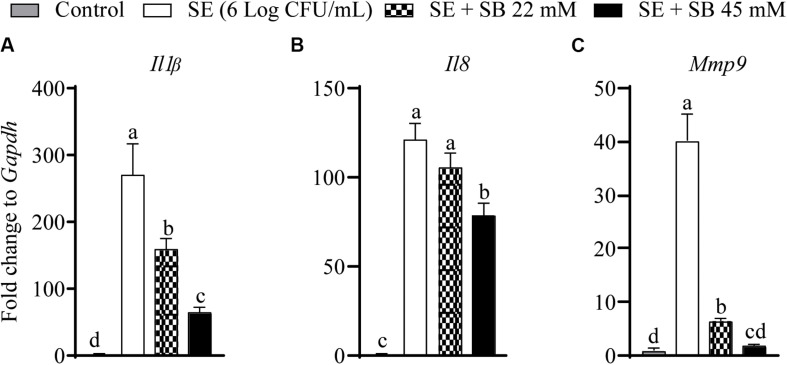
The effect of SICs (22 and 45 mM) of SB on the expression of inflammatory genes [*Il1b* **(A)**, *Il8* **(B)**, *Mmp9* **(C)**] in chicken macrophages challenged with SE. Data presented as fold change normalized to *Gapdh* and error bars represents SEM (*n* = 6). Bars with different letters represents a significant difference at *P* < 0.05.

The effect of sodium butyrate on expression of inflammatory genes (*Il1*β, *Il8*, and *Mmp9*) is shown in Figures 4A–C. The expressions of *Il1*β, *Il8*, and *Mmp9* were significantly up regulated with SE challenge by 328.4, 141.2, and 41.2-fold, respectively, as compared to uninfected chicken macrophages. Presence of 22 mM and 45 mM sodium butyrate reduced *Il1*β gene expression by 41.74 and 76.7%, respectively (*P* < 0.05) (Figure 4A). Similarly, 22 and 45 mM sodium butyrate reduced *Mmp9* gene expression by 84.2 and 95.6%, respectively (*P* < 0.05) (Figure 4C). There was no change in the expression of *Il8* gene after treatment with 22 mM sodium butyrate (*P* > 0.05); however, 45 mM sodium butyrate reduced its expression by 35% (*P* < 0.05) (Figure 4B). Also, there was no significant change on the expression of *Il18, Il10*, *Il6*, *iNos2*, *Il12*β, and *Il12*α after treatment of SE infected chicken macrophages with sodium butyrate 45 mM for 4 h (*P* > 0.05).

## Discussion

Enterocytes play a vital role in the absorption of nutrients and act as a protective barrier against many pathogenic and non-pathogenic microbes present in the intestinal lumen (Chougule et al., 2012; Rath et al., 2018). Primary chicken enterocytes could be considered as an *in vitro* model for screening of various chemicals that influence intestinal physiology and play a crucial role in immunopathology. However, primary chicken enterocytes are not commercially available. Therefore, we developed an *in vitro* cell culture model and tested the response of the primary enterocytes to various chemicals encountered in chicken gut. The enterocytes exhibited epithelial phenotype, were alkaline phosphatase positive, and maintained similar morphologies during successive cultures, tested up to 6–7 passages. The duration to form monolayer ranged between 48 and 96 h. Based on the specialized isolation procedure (superficial scraping of intestinal villi), growth pattern (2–3 days to form monolayer) and morphology (polygonal cells with regular dimensions) of the cells it appeared that the majority of the cells were of epithelial phenotype. A detailed analysis of the primary chicken enterocytes including their cytochemical characterization and response to different chemical stimuli, selected micronutrients, microbial toxins, and metabolic modulators was conducted in our previous publication (Rath et al., 2018). Based on our immunofluorescence staining, the cultured enterocytes were positive for pan Cadherin, actin, Na-K-ATPase, and to a lesser extent for ZO1, a tight junction protein. For this manuscript, we studied the effect of sodium butyrate on the viability and morphological changes in enterocytes before using the cells for experimentation.

Short-chain fatty acids including butyrate are fermentation products of undigested carbohydrates produced in the ceca or colon of animals. Butyrate has been considered as a primary energy source for growth of intestinal epithelial cells (Clausen and Mortensen, 1995; Józefiak et al., 2004) and possess antimicrobial activity against invading pathogens in intestinal lumen (Sunkara et al., 2011; Schulthess et al., 2019). Butyric acid has been Generally Recognized as Safe (GRAS) for its use in foods (Butyric acid- 21CFR182.60, Food and Drug Administration (Food and Drug Administration [FDA], 2017). Butyrate supplementation in the diet of broiler chickens maintains physiological function of intestinal mucosa and gut health (Hu and Guo, 2007; Smulikowska et al., 2009; Wu et al., 2016, 2018). In addition, previous studies also reported that butyrate supplementation in the diet of broiler chickens reduces SE infection (Fernández-Rubio et al., 2009; Cerisuelo et al., 2014; Abd El-Ghany et al., 2016; Arbab et al., 2017; Liu et al., 2017). However, there is limited information on the effect of butyrate on SE virulence, colonization factors and host reponse. Therefore, we tested the anti-virulence, anti-colonization potential of sodium butyrate against SE and investigated the effect of sodium butyrate on host genes participating in inflammatory response. We tested the anti-*Salmonella* potential of sodium butyrate at SICs. These concentrations refer to compound concentrations that do not affect bacterial growth/cell viability but potentially modulate the expression of genes and/or proteins in the host or microbial system (Upadhyay et al., 2012; Upadhyaya et al., 2013, 2015; Wagle et al., 2017; Viedma et al., 2018). Our results suggest that sodium butyrate at SICs does not affect the growth of *Salmonella* and cell viability of primary chicken enterocytes and chicken macrophages (Figure 1) indicating that their application at *SIC* levels is not harmful to the enterocytes. Yan and Ajuwon (2017) also reported that butyrate, at 1 mM, promoted intestinal homeostasis by restoring LPS induced impairment of intestinal barrier in porcine intestinal epithelial cell. Since the concentration tested by Yan and Ajuwon was less than our *SIC*, future research investigating the effect of butyrate at 45 mM on intestinal homeostasis challenged with SE should be conducted. Despite the presence of significant defensive barriers in intestine, SE has developed several strategies to colonize gastrointestinal tract of host and invade the intestinal epithelium (Foley et al., 2013; Lhocine et al., 2015). The SE directly interacts with mucosal barrier to colonize and promote its internalization inside host. Our results from primary chicken enterocytes and chicken macrophages cell culture assay (Figure 2) revealed that sodium butyrate significantly reduced SE adhesion to and invasion of chicken enterocytes (Figures 2A,B) which is critical for colonization in chicken gut and reduced SE invasion of chicken macrophages which helps in systemic spread of the pathogen in chickens (Figure 2D). Van Immerseel et al. (2003) had also reported that incubation of SE with 20 or 30 mM butyrate in Luria Bertoni medium for 4 h reduce invasion of SE in avian intestinal epithelial cells.

Since sodium butyrate was used at *SIC* levels, the reduction in adhesion and invasion observed was not due to a reduction in bacterial number, but probably due to changes in the expression of genes responsible for attachment or invasion. Therefore, we investigated the effect of SICs of sodium butyrate on the expression of SE virulence genes using RT-qPCR. Several genes facilitate the attachment, invasion and translocation of *Salmonella* through the host epithelium by secreting bacterial effector proteins. For example *flgG*, *fimD*, and *prot6E* are genes critical for motility (De Buck et al., 2004; Clavijo et al., 2006; Gantois et al., 2008); whereas *lrp* codes for virulence regulation (Baek et al., 2009); *invH* contributes to invasion (Pati et al., 2013); *sopB*, *sopE*, *sopE2*, *sipB*, *pipB*, *ssaV, orf245, sipA*, *sipC*, *sipD* are critical for type three secretion system function (Raffatellu et al., 2005; Ly and Casanova, 2007; Haraga et al., 2008; Foley et al., 2013; Wemyss and Pearson, 2019). Genes *sodC, spvB*, and *mgtC* facilitate macrophage survFival (Moncrief and Maguire, 1998; Choi et al., 2019), whereas, *rpoS* gene is important for stress tolerance (Shah et al., 2012) and *rfbH* critical for lipopolysaccharide biosynthesis (Gantois et al., 2008). However, the gene expression analysis revealed that there was no effect of SB on the expression of *flgG*, *prot6E*, *invH*, *sipB*, *pipB*, *lrp*, *tatA*, *mrr1*, *spvB*, and *mgtC* (*P* > 0.05) whereas, a few SE genes were upregulated by SB treatment such as *fimD*, *ssaV, orf245, sipA*, *sopB*, *xthA*, *ssrA*, *sodC*, and *rfbH* (Figure 3). Previous investigations by Gantois et al. (2006) has shown that exposure of *Salmonella enterica* serovar Enteritidis and *Salmonella enterica* serovar Typhimurium 10 mM butyrate downregulated the expression of 19 genes encoding for SPI1 effector proteins and invasion including *invF*, *invE*, *invB, pipC*, and *sopB*. Immerseel et al. (2004) had also reported that expression of *hilA* gene associated with invasion of *Salmonella enterica* serovar Enteritidis was reduced after exposure with 2 mM caproic acid, capric acid, and caprylic acid. However, in our study the majority of genes were either not affected or overexpressed. This could be due to differences in the strain variation, time of incubation and other factors.

Since SE gene expression data was not conclusive of the anti-*Salmonella* mechanism of action of butyrate, we investigated the effect of sodium butyrate on host inflammatory response. SE infection damages intestinal mucosal barrier and increases susceptibility to intestinal inflammation, which leads to activation of pro-inflammatory pathways in cells inhabiting submucosal niches. Invasion of *Salmonella* in human intestinal epithelial cells induces release of pro-inflammatory cytokines such as *Il8*, *Il1*β, and *Il18* which further induce secretion of *Il17* and *Il22* and augment inflammatory response in the intestinal mucosa (Royle et al., 2003; Larock et al., 2015; Huang et al., 2020). Previous research from our group has shown that treatment of LPS stimulated chicken macrophages with 1 mM butyrate downregulated expression of inflammatory cytokines such as *Il1*β, *Il6*, *IFN-gamma*, and *Il10* (Zhou et al., 2014). Our results revealed that sodium butyrate significantly reduced inflammatory cytokines such as *Il1*β, *Il8*, and *Mmp9* in chicken macrophages (Figure 4) infected with SE indicating that since butyrate reduced inflammation in chicken macrophages, it could reduce *Salmonella* invasion process. Bedford and Gong (2018) had also described that anti-inflammatory properties of butyrate could be mediated by the reduction of pro-inflammatory cytokine expression such as interferon gamma (*IFN-g*), tumor necrosis factor-α (*TNF-*α), interleukin-1β (*Il1*β), *Il6*, and *Il8*.

In conclusion, our study showed that sodium butyrate, at sub-inhibitory concentration, significantly reduced colonization potential of SE by reducing attachment and invasion capacity on chicken enterocytes and macrophage. Moreover, sodium butyrate exerted its anti-inflammatory effect on chicken macrophages (challenged with SE) by downregulation of inflammatory cytokine genes. Results suggest that sodium butyrate could potentially be used as a safe and effective compound to reduce SE colonization in chickens, however, *in vivo* studies validating the *in vitro* efficacy of sodium butyrate are needed.

## Data Availability Statement

The raw data supporting the conclusions of this article will be made available by the authors, without undue reservation, to any qualified researcher.

## Ethics Statement

This study was reviewed and approved by the Institutional Animal Care and Use Committee, University of Arkansas.

## Author Contributions

AG, AU, and XS designed the experiments. AG, MB, and BW conducted the experiments. AG wrote the manuscript. BW, AU, MB, XS, NR, and AD critically analyzed and revised the manuscript. All authors contributed to the article and approved the submitted version.

## Disclaimer

Mention of a trade name, proprietary product, or specific equipment does not constitute a guarantee or warranty by the USDA and does not imply its approval to the exclusion of other products that may be suitable.

## Conflict of Interest

The authors declare that the research was conducted in the absence of any commercial or financial relationships that could be construed as a potential conflict of interest.

## References

[B1] Abd El-Ghany W. A. Awaad M. H. Nasef S. A. Gaber A. F. (2016). Effect of sodium butyrate on *Salmonella* enteritidis infection in broiler chickens. *Asian J. Poult. Sci.* 10 104–110. 10.3923/ajpsaj.2016.104.110

[B2] Antunes P. Mourão J. Campos J. Peixe L. (2016). Salmonellosis: the role of poultry meat. *Clin. Microbiol. Infect.* 22 110–121. 10.1016/j.cmi.2015.12.004 26708671

[B3] Arbab S. Hafsa Z. Muhammad Y. Saima M. Asim A. Saima A. (2017). Protective effect of sodium butyrate on growth performance, immune responses and gut mucosal morphometry in *Salmonella*-challenged broiler chickens. *Int. J. Agric. Biol.* 19 1387–1393.

[B4] Atterbury R. J. Van Bergen M. A. P. Ortiz F. Lovell M. A. Harris J. A. De Boer A. (2007). Bacteriophage therapy to reduce *Salmonella* colonization of broiler chickens. *Appl. Environ. Microbiol.* 73 4543–4549. 10.1128/AEM.00049-07 17526794 PMC1932804

[B5] Baek C. H. Wang S. Roland K. L. Curtiss R. (2009). Leucine-responsive regulatory protein (Lrp) acts as a virulence repressor in *Salmonella enterica* serovar typhimurium. *J. Bacteriol.* 191 1278–1292. 10.1128/JB.01142-08 19074398 PMC2631999

[B6] Bansal M. Nannapaneni R. Kode D. Chang S. Sharma C. S. McDaniel C. (2019). Rugose morphotype in *Salmonella* Typhimurium and *Salmonella* Heidelberg induced by sequential exposure to subinhibitory sodium hypochlorite aids in biofilm tolerance to lethal sodium hypochlorite on polystyrene and stainless steel surfaces. *Front. Microbiol* 10:2704. 10.3389/fmicb.2019.02704 31827464 PMC6890808

[B7] Bearson S. M. D. Bearson B. L. Sylte M. J. Looft T. Kogut M. H. Cai G. (2019). Cross-protective *Salmonella* vaccine reduces cecal and splenic colonization of multidrug-resistant *Salmonella enterica* serovar Heidelberg. *Vaccine* 37 1255–1259. 10.1016/j.vaccine.2018.12.058 30718082

[B8] Bedford A. Gong J. (2018). Implications of butyrate and its derivatives for gut health and animal production. *Anim. Nutr.* 4 151–159. 10.1016/j.aninu.2017.08.010 30140754 PMC6104520

[B9] Byrd J. A. Hargis B. M. Caldwell D. J. Bailey R. H. Herron K. L. McReynolds J. L. (2001). Effect of lactic acid administration in the drinking water during preslaughter feed withdrawal on *Salmonella* and Campylobacter contamination of broilers. *Poult. Sci.* 80 278–283. 10.1093/ps/80.3.278 11261556

[B10] CDC (2013). *Antibiotic Resistance Threats in the United States.* Available online at: https://www.cdc.gov/drugresistance/threat-report-2013/pdf/ar-threats-2013-508.pdf (accessed January 24, 2020).

[B11] CDC (2018). *Outbreaks Involving Salmonella* | CDC. Available online at: https://www.cdc.gov/salmonella/outbreaks.html (accessed February 4, 2020).

[B12] Cerisuelo A. Marín C. Sánchez-Vizcaíno F. Gómez E. A. De La Fuente J. M. Durán R. (2014). The impact of a specific blend of essential oil components and sodium butyrate in feed on growth performance and *Salmonella* counts in experimentally challenged broilers. *Poult. Sci.* 93 599–606. 10.3382/ps.2013-03528 24604853

[B13] Chadfield M. S. Hinton M. H. (2004). Effects of furazolidone pretreatment of *Salmonella* enteritidis PT4 at sub- and suprainhibitory concentrations on phagocytosis and intracellular survival in chicken macrophages. *Vet. Immunol. Immunopathol.* 100 81–97. 10.1016/j.vetimm.2004.03.004 15182998

[B14] Choi S. Choi E. Cho Y.-J. Nam D. Lee J. Lee E.-J. (2019). The *Salmonella* virulence protein MgtC promotes phosphate uptake inside macrophages. *Nat. Commun* 10 1–14. 10.1038/s41467-019-11318-2 31346161 PMC6658541

[B15] Chougule P. Herlenius G. Hernandez N. M. Patil P. B. Xu B. Sumitran-Holgersson S. (2012). Isolation and characterization of human primary enterocytes from small intestine using a novel method. *Scand. J. Gastroenterol.* 47 1334–1343. 10.3109/00365521.2012.708940 22943429 PMC3490477

[B16] Clausen M. R. Mortensen P. B. (1995). Kinetic studies on colonocyte metabolism of short chain fatty acids and glucose in ulcerative colitis. *Gut* 37 684–689. 10.1136/gut.37.5.684 8549946 PMC1382875

[B17] Clavijo R. I. Loui C. Andersen G. L. Riley L. W. Lu S. (2006). Identification of genes associated with survival of *Salmonella enterica* serovar enteritidis in chicken egg albumen. *Appl. Environ. Microbiol.* 72 1055–1064. 10.1128/AEM.72.2.1055-1064.2006 16461649 PMC1392908

[B18] Darre M. J. Kollanoor-Johny A. Venkitanarayanan K. Upadhyaya I. (2014). Practical implications of plant-derived antimicrobials in poultry diets for the control of *Salmonella* Enteritidis. *J. Appl. Poult. Res.* 23 340–344. 10.3382/japr.2014-00942

[B19] De Buck J. Van Immerseel F. Haesebrouck F. Ducatelle R. (2004). Effect of type 1 fimbriae of *Salmonella enterica* serotype Enteritidis on bacteraemia and reproductive tract infection in laying hens. *Avian. Pathol.* 33 314–320. 10.1080/0307945042000220561 15223560

[B20] Fàbrega A. Vila J. (2013). *Salmonella enterica* serovar Typhimurium skills to succeed in the host: virulence and regulation. *Clin. Microbiol. Rev* 26 308–341. 23554419 10.1128/CMR.00066-12 PMC3623383

[B21] Fernández-Rubio C. Ordóñez C. Abad-González J. Garcia-Gallego A. Honrubia M. P. Mallo J. J. (2009). Butyric acid-based feed additives help protect broiler chickens from *Salmonella* enteritidis infection. *Poult. Sci.* 88 943–948. 10.3382/ps.2008-00484 19359681

[B22] Fiorentin L. Vieira N. D. Barioni W. (2005). Oral treatment with bacteriophages reduces the concentration of *Salmonella* Enteritidis PT4 in caecal contents of broilers. *Avian. Pathol.* 34 258–263. 10.1080/01445340500112157 16191711

[B23] Foley S. L. Johnson T. J. Ricke S. C. Nayak R. Danzeisen J. (2013). *Salmonella* Pathogenicity and Host Adaptation in Chicken-Associated Serovars. *Microbiol. Mol. Biol. Rev.* 77 582–607. 10.1128/mmbr.00015-13 24296573 PMC3973385

[B24] Food and Drug Administration [FDA] (2017). *CFR Code of Federal Regulations Title 21 The information on this page is current as of. (April)*1 2016. Silver Spring, ML: FDA.

[B25] Gantois I. Ducatelle R. Pasmans F. Haesebrouck F. Thompson A. Hinton J. C. (2006). Butyrate specifically down-regulates *Salmonella* pathogenicity island 1 gene expression. *Society* 72 946–949. 10.1128/AEM.72.1.946 PMC1352287 16391141

[B26] Gantois I. Ducatelle R. Pasmans F. Haesebrouck F. Van Immerseel F. (2008). *Salmonella enterica* serovar enteritidis genes induced during oviduct colonization and egg contamination in laying hens. *Appl. Environ. Microbiol.* 74 6616–6622. 10.1128/AEM.01087-08 18776023 PMC2576714

[B27] Gart E. V. Suchodolski J. S. Welsh T. H. Alaniz R. C. Randel R. D. Lawhon S. D. (2016). *Salmonella* typhimurium and multidirectional communication in the gut. *Front. Microbiol.* 7:827. 10.3389/fmicb.2016.01827 27920756 PMC5118420

[B28] Hallstrom K. McCormick B. A. (2011). *Salmonella* interaction with and passage through the intestinal mucosa: through the lens of the organism. *Front. Microbiol.* 2:88. 10.3389/fmicb.2011.00088 21747800 PMC3128981

[B29] Haraga A. Ohlson M. B. Miller S. I. (2008). Salmonellae interplay with host cells. *Nat. Rev. Microbiol.* 6, 53–66. 10.1038/nrmicro1788 18026123

[B30] Hoffmann S. Anekwe T. D. (2015). “Making sense of recent cost-offoodborne-illness estimates,” in *Econ. Cost Foodborne Illnesses United States* (New York: Nova Science Publishers, Inc.), 77–115.

[B31] Hu Z. Guo Y. (2007). Effects of dietary sodium butyrate supplementation on the intestinal morphological structure, absorptive function and gut flora in chickens. *Anim. Feed Sci. Technol.* 132 240–249. 10.1016/j.anifeedsci.2006.03.017

[B32] Huang K. Fresno A. H. Skov S. Olsen J. E. (2020). Dynamics and outcome of macrophage interaction between *Salmonella* gallinarum, *Salmonella* Typhimurium, and *Salmonella* dublin and macrophages from chicken and cattle. *Front. Cell. Infect. Microbiol.* 9:420. 10.3389/fcimb.2019.00420 31998655 PMC6966237

[B33] Immerseel F. V. Buck J. D. Boyen F. Bohez L. Pasmans F. Volf J. (2004). Medium-Chain Fatty Acids Decrease Colonization and Invasion through hilA Suppression Shortly after Infection of Chickens with *Salmonella enterica* Serovar Enteritidis. *Appl. Environ. Microbiol.* 70 3582–3587. 10.1128/aem.70.6.3582-3587.2004 15184160 PMC427757

[B34] Inoue A. Y. Berchieri A. Bernardino A. Paiva J. B. Sterzo E. V. (2008). Passive Immunity of Progeny from Broiler Breeders Vaccinated with Oil-Emulsion Bacterin Against *Salmonella* Enteritidis. *Avian. Dis.* 52 567–571. 10.1637/8096-082707-reg.1 19166046

[B35] Johny A. K. Frye J. G. Donoghue A. Donoghue D. J. Porwollik S. McClelland M. (2017). Gene expression response of *Salmonella enterica* serotype enteritidis phage type 8 to subinhibitory concentrations of the plant-derived compounds trans-cinnamaldehyde and eugenol. *Front. Microbiol.* 8:828. 10.3389/fmicb.2017.01828 29018419 PMC5623010

[B36] Józefiak D. Rutkowski A. Martin S. A. (2004). Carbohydrate fermentation in the avian ceca: a review. *Anim. Feed Sci. Technol.* 113 1–15. 10.1016/j.anifeedsci.2003.09.007

[B37] Jung J. W. Cho S. D. Ahn N. S. Yang S. R. Park J. S. Jo E. H. (2005). Ras/MAP kinase pathways are involved in Ras specific apoptosis induced by sodium butyrate. *Cancer Lett* 225 199–206. 10.1016/j.canlet.2004.11.029 15978324

[B38] Kauber K. Fowler H. Lipton B. Meschke J. S. Rabinowitz P. (2017). *Salmonella* knowledge. attitudes and practices: a survey of backyard poultry owners residing in seattle, washington and the surrounding metropolitan area. *Zoonoses Public Health* 64 21–28. 10.1111/zph.12274 27329695 PMC5179318

[B39] Kim W. I. Choi S. Y. Han I. Cho S. K. Lee Y. Kim S. (2020). Inhibition of *Salmonella enterica* growth by competitive exclusion during early alfalfa sprout development using a seed-dwelling *Erwinia persicina* strain EUS78. *Int. J. Food Microbiol.* 312:108374. 10.1016/j.ijfoodmicro.2019.108374 31669765

[B40] Kohli N. Crisp Z. Riordan R. Li M. Alaniz R. C. Jayaraman A. (2018). The microbiota metabolite indole inhibits *Salmonella* virulence: involvement of the PhoPQ two-component system. *PLoS One* 13:e0190613. 10.1371/journal.pone.0190613 29342189 PMC5771565

[B41] Kollanoor-Johny A. Mattson T. Baskaran S. A. Amalaradjou M. A. Babapoor S. March B. (2012). Reduction of *Salmonella enterica* serovar enteritidis colonization in 20-day-old broiler chickens by the plant-derived compounds trans-cinnamaldehyde and eugenol. *Appl. Environ. Microbiol.* 78 2981–2987. 10.1128/AEM.07643-11 22327574 PMC3318785

[B42] Larock D. L. Chaudhary A. Miller S. I. (2015). *Salmonella*e interactions with host processes. *Nat. Rev. Microbiol.* 13 191–205. 10.1038/nrmicro3420 25749450 PMC5074537

[B43] Lhocine N. Arena E. T. Bomme P. Ubelmann F. Prévost M. C. Robine S. (2015). Apical invasion of intestinal epithelial cells by *salmonella* typhimurium requires villin to remodel the brush border actin cytoskeleton. *Cell Host Microbe* 17 164–177. 10.1016/j.chom.2014.12.003 25600187 PMC4346658

[B44] Liu J. D. Bayir H. O. Cosby D. E. Cox N. A. Williams S. M. Fowler J. (2017). Evaluation of encapsulated sodium butyrate on growth performance, energy digestibility, gut development, and *Salmonella* colonization in broilers. *Poult. Sci.* 96 3638–3644. 10.3382/ps/pex174 28938774

[B45] Ly K. T. Casanova J. E. (2007). Mechanisms of *Salmonella* entry into host cells. *Cell. Microbiol.* 9 2103–2111. 10.1111/j.1462-5822.2007.00992.x 17593246

[B46] Marder E. P. Griffin P. M. Cieslak P. R. Dunn J. Hurd S. Jervis R. (2018). Preliminary incidence and trends of infections with pathogens transmitted commonly through food - foodborne diseases active surveillance network, 10 U.S. *sites*, 2006-2017. *Morb. Mortal. Wkly. Rep.* 67 324–328. 10.15585/mmwr.mm6711a3 29565841 PMC5868202

[B47] Moncrief M. B. C. Maguire M. E. (1998). Magnesium and the role of mgtC in growth of *Salmonella* typhimurium. *Infect. Immun.* 66 3802–3809. 10.1128/iai.66.8.3802-3809.1998 9673265 PMC108421

[B48] Nabil N. M. Tawakol M. M. Hassan H. M. (2018). Assessing the impact of bacteriophages in the treatment of *Salmonella* in broiler chickens. *Infect. Ecol. Epidemiol* 8:1539056. 10.1080/20008686.2018.1539056 30397428 PMC6211228

[B49] Nair D. V. T. Venkitanarayanan K. Johny A. K. (2018). Antibiotic-resistant *Salmonella* in the food supply and the potential role of antibiotic alternatives for control. *Foods* 7:167. 10.3390/foods7100167 30314348 PMC6210005

[B50] Okamura M. Lillehoj H. S. Raybourne R. B. Babu U. S. Heckert R. A. Tani H. (2005). Differential responses of macrophages to *Salmonella enterica* serovars Enteritidis and Typhimurium. *Vet. Immunol. Immunopathol.* 107 327–335. 10.1016/j.vetimm.2005.05.009 16023220

[B51] Pal S. Dey S. Batabyal K. Banerjee A. Joardar S. N. Samanta I. (2017). Characterization of *Salmonella* Gallinarum isolates from backyard poultry by polymerase chain reaction detection of invasion (invA) and *Salmonella* plasmid virulence (spvC) genes. *Vet. World* 10 814–817. 10.14202/vetworld.2017.814-817 28831228 PMC5553153

[B52] Pati N. B. Vishwakarma V. Jaiswal S. Periaswamy B. Hardt W. D. Suar M. (2013). Deletion of invH gene in *Salmonella enterica* serovar Typhimurium limits the secretion of Sip effector proteins. *Microbes Infect.* 15 66–73. 10.1016/j.micinf.2012.10.014 23159244

[B53] Raffatellu M. Wilson R. P. Chessa D. Andrews-Polymenis H. Tran Q. T. Lawhon S. (2005). SipA, SopA, SopB, SopD, and SopE2 contribute to *Salmonella enterica* Serotype Typhimurium invasion of epithelial cells. *Infect. Immun.* 73 146–154. 10.1128/IAI.73.1.146-154.2005 15618149 PMC538951

[B54] Rath N. C. Liyanage R. Gupta A. Packialakshmi B. Lay J. O. (2018). A method to culture chicken enterocytes and their characterization. *Poult. Sci.* 97 4040–4047. 10.3382/ps/pey248 29917122

[B55] Rath N. C. Parcells M. S. Xie H. Santin E. (2003). Characterization of a spontaneously transformed chicken mononuclear cell line. *Vet. Immunol. Immunopathol.* 96 93–104. 10.1016/S0165-2427(03)00143-0 14522138

[B56] Revolledo L. Ferreira A. J. P. Mead G. C. (2006). Prospects in *Salmonella* control: competitive exclusion, probiotics, and enhancement of avian intestinal immunity. *J. Appl. Poult. Res.* 15 341–351. 10.1093/japr/15.2.341

[B57] Royle M. C. J. Tötemeyer S. Alldridge L. C. Maskell D. J. Bryant C. E. (2003). Stimulation of toll-like receptor 4 by Lipopolysaccharide during cellular invasion by live *Salmonella* typhimurium is a critical but not exclusive event leading to macrophage responses. *J. Immunol.* 170 5445–5454. 10.4049/jimmunol.170.11.5445 12759420

[B58] Sakurazawa T. Ohkusa T. (2005). Cytotoxicity of organic acids produced by anaerobic intestinal bacteria on cultured epithelial cells. *J. Gastroenterol.* 40 600–609. 10.1007/s00535-005-1594-z 16007394

[B59] Schulthess J. Pandey S. Capitani M. Rue-Albrecht K. C. Arnold I. Franchini F. (2019). The short chain fatty acid butyrate imprints an antimicrobial program in macrophages. *Immunity* 50 432.e5–445.e5. 10.1016/j.immuni.2018.12.018 30683619 PMC6382411

[B60] Shah D. H. Casavant C. Hawley Q. Addwebi T. Call D. R. Guard J. (2012). *Salmonella* enteritidis strains from poultry exhibit differential responses to acid stress, oxidative stress, and survival in the egg albumen. *Foodborne Pathog. Dis.* 9 258–264. 10.1089/fpd.2011.1009 22304629 PMC3326446

[B61] Sheela R. R. Babu U. Mu J. Elankumaran S. Bautista D. A. Raybourne R. B. (2003). Immune responses against *Salmonella enterica* serovar enteritidis infection in virally immunosuppressed chickens. *Clin. Diagn. Lab. Immunol.* 10 670–679. 10.1128/CDLI.10.4.670-679.2003 12853403 PMC164247

[B62] Smulikowska S. Czerwinski J. Mieczkowska A. Jankowiak J. (2009). The effect of fat-coated organic acid salts and a feed enzyme on growth performance, nutrient utilization, microflora activity, and morphology of the small intestine in broiler chickens. *J. Anim. Feed Sci.* 18 478–489. 10.22358/jafs/66422/2009

[B63] Stern N. J. Cox N. A. Bailey J. S. Berrang M. E. Musgrove M. T. (2001). Comparison of mucosal competitive exclusion and competitive exclusion treatment to reduce *Salmonella* and Campylobacter spp. colonization in broiler chickens. *Poult. Sci.* 80 156–160. 10.1093/ps/80.2.156 11233003

[B64] Sun X. Jia Z. (2018). Microbiome modulates intestinal homeostasis against inflammatory diseases. *Vet. Immunol. Immunopathol.* 205 97–105. 10.1016/j.vetimm.2018.10.014 30459007

[B65] Sun X. Jobin C. (2014). Nucleotide-binding oligomerization domain-containing protein 2 controls host response to Campylobacter jejuni in Il10-/- mice. *J. Infect. Dis.* 210 1145–1154. 10.1093/infdis/jiu148 24620022 PMC4168300

[B66] Sunkara L. T. Achanta M. Schreiber N. B. Bommineni Y. R. Dai G. Jiang W. (2011). Butyrate enhances disease resistance of chickens by inducing antimicrobial host defense peptide gene expression. *PLoS One* 6:e27225. 10.1371/journal.pone.0027225 22073293 PMC3208584

[B67] Upadhyay A. Johny A. K. Amalaradjou M. A. R. Ananda Baskaran S. Kim K. S. Venkitanarayanan K. (2012). Plant-derived antimicrobials reduce Listeria monocytogenes virulence factors in vitro, and down-regulate expression of virulence genes. *Int. J. Food Microbiol.* 157 88–94. 10.1016/j.ijfoodmicro.2012.04.018 22608657

[B68] Upadhyaya I. (2015). *Investigating the Efficacy of Natural Antimicrobial Molecules in Reducing Egg-borne Transmission of Salmonella enterica* serovar Enteritidis in Layer Hens. Available online at: https://opencommons.uconn.edu/dissertations/655 (accessed January 29, 2020).

[B69] Upadhyaya I. Upadhyay A. Kollanoor-Johny A. Darre M. J. Venkitanarayanan K. (2013). Effect of plant derived antimicrobials on *salmonella* enteritidis adhesion to and invasion of primary chicken oviduct epithelial cells in vitro and virulence gene expression. *Int. J. Mol. Sci.* 14 10608–10625. 10.3390/ijms140510608 23698782 PMC3676857

[B70] Upadhyaya I. Upadhyay A. Kollanoor-Johny A. Mooyottu S. Baskaran S. A. Yin H. B. (2015). In-feed supplementation of trans-cinnamaldehyde reduces layer- chicken egg-borne transmission of *Salmonella enterica* serovar enteritidis. *Appl. Environ. Microbiol.* 81 2985–2994. 10.1128/AEM.03809-14 25710365 PMC4393446

[B71] Van Immerseel F. Boyen F. Gantois I. Timbermont L. Bohez L. Pasmans F. (2005). Supplementation of coated butyric acid in the feed reduces colonization and shedding of *Salmonella* in poultry. *Poult. Sci.* 84 1851–1856. 10.1093/ps/84.12.1851 16479940

[B72] Van Immerseel F. De Buck J. Pasmans F. Velge P. Bottreau E. Fievez V. (2003). Invasion of *Salmonella* enteritidis in avian intestinal epithelial cells in vitro is influenced by short-chain fatty acids. *Int. J. Food Microbiol.* 85 237–248. 10.1016/S0168-1605(02)00542-1 12878382

[B73] Viedma E. Pérez-Montarelo D. Villa J. Muñoz-Gallego I. Larrosa N. Fernández-Hidalgo N. (2018). Sub-inhibitory concentrations of oxacillin modify the expression of agr locus in Staphylococcus aureus clinical strains belonging to different clonal complexes. *BMC Infect. Dis.* 18:177. 10.1186/s12879-018-3088-7 29661157 PMC5902860

[B74] Wagle B. R. Upadhyay A. Arsi K. Shrestha S. Venkitanarayanan K. Donoghue A. M. (2017). Application of β-resorcylic acid as potential antimicrobial feed additive to reduce Campylobacter colonization in broiler chickens. *Front. Microbiol* 8:599. 10.3389/fmicb.2017.00599 28428779 PMC5382206

[B75] Wemyss M. A. Pearson J. S. (2019). Host Cell Death Responses to Non-typhoidal *Salmonella* Infection. *Front. Immunol.* 10:758. 10.3389/fimmu.2019.01758 31402916 PMC6676415

[B76] Wilde S. Jiang Y. Tafoya A. M. Horsman J. Yousif M. Vazquez L. A. (2019). *Salmonella*-vectored vaccine delivering three Clostridium perfringens antigens protects poultry against necrotic enteritis. *PLoS One* 14:e0197721. 10.1371/journal.pone.0197721 30753181 PMC6372158

[B77] Wu W. Xiao Z. An W. Dong Y. Zhang B. (2018). Dietary sodium butyrate improves intestinal development and function by modulating the microbial community in broilers. *PLoS One* 13:e0197762. 10.1371/journal.pone.0197762 29795613 PMC5967726

[B78] Wu Y. Zhou Y. Lu C. Ahmad H. Zhang H. He J. (2016). Influence of Butyrate Loaded Clinoptilolite Dietary Supplementation on Growth Performance, Development of Intestine and Antioxidant Capacity in Broiler Chickens. *PLoS One* 11:e0154410. 10.1371/journal.pone.0154410 27104860 PMC4841535

[B79] Xiong H. Guo B. Gan Z. Song D. Lu Z. Yi H. (2016). Butyrate upregulates endogenous host defense peptides to enhance disease resistance in piglets via histone deacetylase inhibition. *Sci. Rep.* 6 1–12. 10.1038/srep27070 27230284 PMC4882515

[B80] Yan H. Ajuwon K. M. (2017). Butyrate modifies intestinal barrier function in IPEC-J2 cells through a selective upregulation of tight junction proteins and activation of the Akt signaling pathway. *PLoS One* 12:e0179586. 10.1371/journal.pone.0179586 28654658 PMC5487041

[B81] Zhou Z. Y. Packialakshmi B. Makkar S. K. Dridi S. Rath N. C. (2014). Effect of butyrate on immune response of a chicken macrophage cell line. *Vet. Immunol. Immunopathol.* 162 24–32. 10.1016/j.vetimm.2014.09.002 25278494

